# Investigation on Carbohydrate Counting Method in Type 1 Diabetic Patients

**DOI:** 10.1155/2014/176564

**Published:** 2014-08-17

**Authors:** Osman Son, Belgin Efe, Nazan Erenoğlu Son, Aysen Akalin, Nur Kebapçi

**Affiliations:** ^1^Private Sakarya Hospital, Endocrinology Department, Eskisehir, Turkey; ^2^Medical Faculty, Endocrinology Department, Eskisehir Osmangazi University, Eskisehir, Turkey; ^3^Medical Faculty, Diet Department, Eskisehir Osmangazi University, Eskisehir, Turkey

## Abstract

*Objective*. The results from Diabetes Control and Complications Trial (DCCT) have propounded the importance of the approach of treatment by medical nutrition when treating diabetes mellitus (DM). During this study, we tried to inquire carbohydrate (Kh) count method's positive effects on the type 1 DM treatment's success as well as on the life quality of the patients. *Methods*. 22 of 37 type 1 DM patients who applied to Eskişehir Osmangazi University, Faculty of Medicine Hospital, Department of Endocrinology and Metabolism, had been treated by Kh count method and 15 of them are treated by multiple dosage intensive insulin treatment with applying standard diabetic diet as a control group and both of groups were under close follow-up for 6 months. Required approval was taken from the Ethical Committee of Eskişehir Osmangazi University, Medical Faculty, as well as informed consent from the patients. The body weight of patients who are treated by carbohydrate count method and multiple dosage intensive insulin treatment during the study beginning and after 6-month term, body mass index, and body compositions are analyzed. A short life quality and medical research survey applied. At statistical analysis, *t*-test, chi-squared test, and Mann-Whitney *U* test were used. *Results*. There had been no significant change determined at glycemic control indicators between the Kh counting group and the standard diabetic diet and multiple dosage insulin treatment group in our study. *Conclusion*. As a result, Kh counting method which offers a flexible nutrition plan to diabetic individuals is a functional method.

## 1. Introduction 

The importance of maintaining a strict glycemic control in diabetics is well-established. To achieve the desired targets, medical diet therapy, exercise, and the medical strategies should be administered accurately and regularly. The results from the DCCT have demonstrated the importance of medical diet therapy in the treatment of diabetes mellitus (DM). One of the diet strategies recommended by the DCCT is the carbohydrate counting method, which has started to draw attention in the recent years [1–7]. As the diabetes mellitus is a chronic disease which effects quality life and the mental status of patients, it is important to improve the quality life of such patients and to pay attention to the life style changes [8, 9].

Counting carbohydrates leans upon 3 basic facts.

1. Clinical studies have shown that carbohydrates are the main factor that effects the postprandial blood glucose level and determines the need of insulin.
2. Carbohydrates are transformed into glucose in 2 hours after the ingestion and they get into systemic circulation from the first 15 min.
3. Postprandial glycemic response and need of insulin levels are determined by total carbohydrate amount that is ingested rather than the kind of carbohydrate [3–7].

The present study is designed to investigate the effects of the carbohydrate counting method, a medical diet strategy, on the quality of life as well as the success of the treatment in type 1 diabetic patients.

## 2. Materials and Methods

Thirty-seven patients with DM type 1 who were under surveillance in Eskişehir Osmangazi University, Medical Faculty, Department of Internal Diseases, Field of Endocrinology and Metabolic Diseases, Diabetes Outpatient Department, were included in the study. Kh counting method was applied on 22 patients who contented to experience this method and the other 15 cases had multiple dose intensive insulin treatment and standard diabetic diet as control group, and these two groups were closely followed up. Required approval was taken from the Ethical Committee of Eskişehir Osmangazi University, Medical Faculty, as well as informed consent from the patients. Required approval was taken from the Ethical Committee of Eskişehir Osmangazi University, Medical Faculty, as well as informed consent from the patients. Twenty-two patients were given Kh counting method training by a dietitian in three steps mentioned below. At Level 1, Kh counting method, its pros, and cons were explained to the patients. In the second step, the patients were asked about their opinions about Kh counting method and their questions were answered. At Level 2, the patients were informed about how much carbohydrate exists in which food, the effects of the changes in food preparation, and the effects of protein, fiber, and fat on carbohydrate absorption. Moreover, in this period we tried to find out the carbohydrate amount in various food portions using measuring cups. At Level 3, the patients who were familiar with Kh counting method were admitted to the hospital and first application was started accompanied by a dietitian. In this period, other than various food groups the patients were made to practice the method according to their food preferences. Besides, conformity of the method practiced with the insulin used was assessed. The patients were made to stay at the hospital until they could apply the method on their own. This period was found as 7 days according to the learning capacities of the patients. Furthermore, their glycemic indices in their normal life routine were evaluated once every 3 days. The patients were monitored in continuous communication with the doctor and the dietitian from whom they learned the Kh counting method. Diabetes years of the patients involved in the study were recorded from their diabetes files. In the first examination and in the examination after 6 months of the 22 patients practicing carbohydrate counting and 15 patients defined as control group all with Type 1 DM, the following data were reported. Height and weight of the cases were recorded with the same standard measurement device. Systolic and diastolic blood pressure (mmHg) of the patients were recorded with the same tension gauge while being seated after 15 minutes of rest. Waist and hip circumference of the patients were recorded in centimeters. Waist/hip ratio was calculated by dividing waist circumference (cm) into hip circumference (cm). Moreover, BMI (body mass index), FAT% (fat ratio), fat mass, and FFM (fatless mass) ve TBW (total body water) of the patients were measured by using Body Composition Analyzer (TBF-300 M) device. Preprandial morning venous blood samples of the patients after around 10 hours were taken and HbA1c and fructosamine levels were measured using Roche/911 Hitachi device and proper (modular p) kit. Average of three-day preprandial and postprandial blood glucose (mg/dL) values, total cholesterol (mg/dL), triglyceride (mg/dL), and HDL cholesterol (mg/dL) were measured using immunometric chemiluminescence method and Immulite∖1000 device. LDL cholesterol value was calculated with Friedewald formula. LDL cholesterol = total cholesterol – (HDL + triglyceride/5). Total cholesterol/HDL ratio was calculated. The patients were assessed for diabetic retinopathy and nephropathy at the beginning of the study and in the 6th month of the study by means of fundoscopy and GFR (glomerular filtration rate) and of their microalbuminuria levels. Daily total insulin doses of the patients in the carbohydrate counting group and in the control group were recorded before and 6 months after the study. Major hypoglycemia attack frequency (symptomatic and/or blood glycose value below 50 mg/dL) doses of the patients in the carbohydrate counting group and in the control group were recorded before and 6 months after the study of the living quality of the patients in the carbohydrate counting group and in the control group were assessed before and 6 months after the study using Turkish version of a 36-item short health survey and living quality scale* (Medical Outcomes Study 36-Item Short Form (MOS SF- 36))*. With this scale living quality was assessed taking 9 functions into account. These are general state of health, change in health over the last one year, physical function, mental function, social function, pain, mental health, and energy [10, 11]. Results were given as mean ± standard error using parametric tests for the variables showing a homogenous distribution. For the variables not showing a homogenous distribution it was defined as median using nonparametric tests. The difference between the values at the beginning of the study and in the 6th month of the study was calculated with* t*-test and the difference between nonparametric values with chi-squared test and Mann-Whitney* U* test. A total of 37 type 1 diabetic patients under follow-up at the Eskisehir Osmangazi Medical Faculty, Internal Diseases Department, Endocrinology and Metabolism Section, Diabetes Polyclinic, were included in the trial. 22 patients volunteering to apply the carbohydrate counting method were administered with this method while the remaining 15 patients received multiple dose intensive insulin treatment and standard diabetic diet and kept under close monitoring for 6 months.

## 3. Results

22 patients were trained on the carbohydrate counting method by the dietician. Patients' characteristics were summarized in Table 1.

Patients were monitored for 6 months. While one of the patients using the carbohydrate counting method continuously used aspart insulin via insulin infusion pump, all the other patients were administered with multiple dose insulin injection. Prior to treatment, patients were using NPH or insulin glargine as basal insulin and short acting (regular) or fast-acting (aspart insulin) insulin as bolus. In patients using the carbohydrate counting method, insulin glargine was initiated as the basal insulin on the day they shifted to this method (initiated as 40% of the pretreatment total insulin dose and dose adjusted according to patient's requirements) and fast-acting aspart insulin as the bolus while patients in the control group maintained their current pretreatment insulin.

The group of patients applying the carbohydrate counting method and the control group were observed not exhibiting a statistically significant difference in age and diabetes duration (*P* > 0.05) and having similar characteristics.

At study baseline, there was no statistically significant difference between the carbohydrate counting group and the control group with respect to systolic and diastolic blood pressure, fasting and postprandial blood glucose, HbA1c, fructosamine, uric acid, triglyceride, total cholesterol, LDL cholesterol, and total cholesterol/HDL ratio (*P* > 0.05). The baseline HDL level was higher in the carbohydrate counting group (*P* < 0.05*).

At 6 months of the trial, an increase was detected in both systolic and diastolic blood pressure in the carbohydrate counting group. Although these increases remained within the normal limits, there was no statistical significance for systolic blood pressure while the increase in the diastolic blood pressure was considerable (*P* < 0.01**).

In the carbohydrate counting group, there was no statistically significant difference in fasting and postprandial blood glucose, HbA1c, and fructosamine levels at 6 months of treatment compared to the study baseline (*P* > 0.05).

In the carbohydrate counting group, total cholesterol, triglyceride, and HDL cholesterol values were decreased and a statistically nonsignificant increase was detected in LDL cholesterol and total cholesterol/HDL cholesterol ratios at the end of the 6th month. In the 6-month period, a statistically considerable increase was detected in the uric acid values (*P* < 0.01**) (Table 2).

In the control group, there was no statistically significant difference in systolic and diastolic blood pressure, fasting and postprandial blood glucose, HbA1c, fructosamine, uric acid, triglyceride, total cholesterol, LDL cholesterol, and total cholesterol/HDL ratio at the end of 6 months compared to study baseline (*P* > 0.05).

No statistically significant difference was detected between the carbohydrate counting group and the control group with respect to diabetic retinopathy findings, microalbuminuria, GFR, and hypoglycemia frequency markers (*P* > 0.05) at study baseline. There were no findings of macrovascular complications in either group.

There was no change detected in findings of diabetic retinopathy in the carbohydrate counting group and the control group. Despite the reduction observed in the frequency of microalbuminuria and hypoglycemia in the carbohydrate counting group, this did not reach a level of statistical significance (*P* > 0.05).

In the control group, values for microalbuminuria, GFR, and hypoglycemia frequency showed a statistically nonsignificant increase at the end of 6 months compared to study baseline (*P* > 0.05).

At 6 months of treatment, hypoglycemia frequency was detected to be decreased in the carbohydrate counting group while it increased in the control group. However these values were not statistically significant (*P* > 0.05). In addition, upon comparison of the change in the hypoglycemia frequency between the two groups, the difference was not detected to be statistically significant (Mann-Whitney* U* test *i*: 0.138 *P* > 0.05).

At study baseline, no marked difference was detected in the total insulin doses between the carbohydrate counting group and the control group (*P* > 0.05). In the carbohydrate counting group, there was a statistically significant reduction in the total insulin dose at 6 months compared to baseline (55.22 ± 4.70 IU versus 43.77 ± 3.05, *P* < 0.004). In the control group, total insulin doses were also detected to be decreased at the end of 6 months compared to study baseline; however this reduction did not reach statistical significance (*P* > 0.05) (Table 3).

Comparison of the body composition findings in the carbohydrate counting group prior to trial and at 6 months of the trial revealed the following results: there was no statistically significant difference in waist circumference, body weight, waist/hip ratio, and BMI values at 6 months compared to baseline (*P* > 0.05). While a statistically significant reduction was detected in the fat mass and %FAT values, a significant increase was detected in the FFM and TBW values (*P* < 0.05*).

In the carbohydrate counting group, comparison of the baseline quality of life data with the 6-month data revealed a favorable trend in all variables indicating an increased quality of life except for pain. The favorable changes in overall health and the change in health status within the last one year (*P* < 0.01**), physical function, mental function, social function and energy (*P* < 0.05*) were detected to be statistically significant (Table 4).

In the control group, the comparison of the baseline quality of life data with the 6-month data revealed a statistically significant increase only in overall health scores (general point of view: 47.66 ± 5.27 versus 56.06 ± 3.97, *P* = 0.015) (*P* < 0.05*). There was no statistically significant change in the other values (*P* > 0.05) (Table 5).

As for the assessments of the physical function, physical functionality, mental function, and social function, the statistically significant favorable change is more marked in the carbohydrate counting group compared to the control group (*P* < 0.05*). There was no statistically significant difference between the two groups in pain, mental health, and energy level (*P* > 0.05).

## 4. Discussion

The primary targets of the diabetes treatment include maintenance of life, reduction of symptoms, and increase of the quality of life. Secondarily, the treatment is aimed at the prevention of the long-term chronic complications and early mortality [1–7, 12].

Although both systolic and diastolic blood pressure were at the recommended values in our study, diastolic blood pressure levels were higher in the carbohydrate counting group at the controls performed 6 months later relative to study baseline. This may be attributed to the excessive salt intake resulting from the lack of constraint in diet although salt intake was not recorded in the patients.

Since diabetes mellitus is a disease that affects the carbohydrate, protein, and fat metabolism, nutrition should always be included in the diabetes treatment and training programs [3–9]. Recently, the interest in medical diet therapy has increased in the treatment of DM because medical diet therapy in diabetic individuals was demonstrated to indicate an improved glycemic control as confirmed by an approximate reduction of 1-2 units in HbA1c [3–9, 12, 13].

The trials have suggested that the total amount of carbohydrate intake is more important than the type and source of carbohydrates taken during the main and intermediate meals in type 1 and type 2 DM [3–13].

In type 1 diabetic patients, the lipid profile observed along with the high blood glucose level includes hypertriglyceridemia and HDL lowness, which may be corrected by active insulin treatment [2, 14].

In our study, HDL and triglyceride values were decreased to some extent while LDL cholesterol, total cholesterol/HDL ratio, and the uric acid level were increased. As for the control group, there was no significant change detected. Carbohydrate counting method is beneficial for motivated patients in whom this method can be administered by dieticians. However while applying this method that provides flexibility in eating, patients focus on a macronutrient. Some patients may deviate from their normal diet regimen along with excessive daily energy intake. Therefore, the importance of protein and fat intake should also be adequately explained to the patients applying the carbohydrate counting methods as well as the carbohydrate values. Carbohydrate counting method should be considered in the context of fundamental healthy nutrition plan [2–15].

The association of type 2 DM with macrovascular complications is well-established. However in type 1 diabetic patients, the incidence of macrovascular complications will increase along with the duration of diabetes. In this study, no findings of macrovascular complication were detected in the carbohydrate counting group or the control group. This situation may be related to the young age and the short duration of diabetes observed in our patients.

In patients with a long history of diabetes, the risk of developing both microvascular and macrovascular disease is high. In the UKPDS trial, the importance of a strict blood pressure control as well as a strict blood glucose control in prevention of diabetic complications in type 2 diabetes has been demonstrated while the DCCT study showed the importance of a strict blood glucose control in type 1 diabetes [16–19].

In type 1 diabetic patients, intensive treatment delays the onset of clinically important retinopathy, nephropathy, and neuropathy and slows down the progression by 30–75% [17].

In our study, microalbuminuria values were observed to be decreased to some extent in the carbohydrate counting group while they were detected to be increased in the control group. However these changes did not reach a level of significance. In the DCCT trial, intensive insulin treatment decreased the risk of albuminuria and microalbuminuria by 54% and 39%, respectively [16–18].

If not hindered by hypoglycemia, the HbA1c levels of all diabetic patients would be normal throughout life [10, 20–24]. Hypoglycemia limits the long-term benefits of glycemic control in type 1 diabetes.

In studies where the prandial (bolus) insulin dose is adjusted according to the total carbohydrate content of the meal (or the intermediate meal), the HbA1c level is reported to be decreased by 1–1.5 units [23].

Hypoglycemia frequency was detected to decrease in the carbohydrate counting group while it increased in the intensive treatment control group compared to baseline. However these changes were not statistically significant. At 6 months, there was a favorable difference in the carbohydrate counting group with respect to hypoglycemia frequency; however this difference was not statistically significant compared to the control group.

One of the potential problems that may be experienced by patients applying the carbohydrate counting method is the increased food consumption due to the lack of restriction applied in patients and thus the increase in daily insulin amount administered. Among the patients participating in our trial, the total insulin doses statistically significantly decreased 6 months later compared to baseline in the carbohydrate counting group contrary to what is feared. As for the patients in the control group, the total daily insulin dose decreased; however this reduction did not reach a level of significance.

Weight gain represents a major issue in diabetic patients. In type 1 diabetes, weight gain may result from the imbalance in nutritional factors, physical inactivity, or increased food consumption due to frequent hypoglycemic attacks.

When the carbohydrate counting method is to be applied, the other macronutrients such as fat and protein and their intake amount should be taken into consideration [6–10, 12, 13, 24].

In the carbohydrate counting method, focus is given on only one macronutrient. Patients only count the carbohydrate in their food. In addition, there is no fixed calorie limitation in their diet. Accordingly, patients may deviate from their regular nutritional regimen. In addition, these patients have the tendency to maintain their habits of intermediate meals. Therefore, patients should also pay attention to their daily energy, fat, and protein intake as well as the carbohydrate intake [25].

In our study, there was no difference in body weight, BMI, waist circumference, or waist/hip ratio at the 6-month evaluation in the carbohydrate counting group. However contrary to what is feared, a statistically significant reduction was detected in fat mass and % FAT values in patients applying this method. As for the patients in the control group, there was no significant difference in these parameters at 6 months compared to baseline. These results suggest that, when applied to motivated patients, carbohydrate counting method may prevent excessive food consumption via prevention of frequent hypoglycemic attacks, thereby contributing also to weight loss.

Throughout life, it is very important to detect the quality of life in individuals in cases of disease [26]. Despite the presence of active medical therapies in diabetic patients, most of them do not have a good health and life quality. Most of the diabetic patients worry about their life continuously getting worse. Therefore, while presenting a new offer to a diabetic patient on his/her disease, empathy and active communication should be established with the patients and participation of the patient in this organization should be ensured [27].

Diabetes is a disease that progresses primarily with physical and psychological problems and impairs the associated quality of life significantly. The underlying acute and chronic complications affect the quality of life. Social status, level of education, perception of the disease, diabetes-related diet, exercise, and treatment protocol affect the quality of life as well as the glycemic control in diabetic patients.

In our study, the baseline parameter of social function was detected to be higher in the carbohydrate counting method compared to the control group on quality of life scales. This has demonstrated the importance of the social status of patients in perceiving their disease and the carbohydrate counting method. Therefore, this method is only appropriate for a selected motivated group of patients with a high perception [28–34].

Carbohydrate counting is a method that provides flexibility and increases quality of life within the nutritional regimen in voluntary patients. The improvement observed in the short term is reported to be maintained also in the long term. In the trials performed, the strategy based on adjustment of the insulin dose according to the carbohydrate content of the meal is suggested to be more successful compared to low glycemic index diet in type 1 diabetics. This approach enables lack of constraint in eating and selection of food for diabetic individuals while maintaining glycemic control [35–39].

In type 1 diabetes, achievement of success was demonstrated by teaching of the way to adjust blood glucose as well as the lack of constraint in eating offered to patients. While a significant improvement was demonstrated in HbA1c level in this patient group, there was no significant increase in severe hypoglycemia. What is more, the quality of life was increased by this method; tolerability of treatment and psychological well-being were observed despite the increase in the number of injections and blood glucose monitorization.

In our study, the quality of life was detected to be increased in the carbohydrate counting group as compared to the control group and the pretrial period.

*In conclusion,* the carbohydrate counting method is not a new approach. There are data indicating that this method has been applied in the nutritional plan of the diabetic patients since 1921 when insulin was discovered. However recently, the interest in this method has increased along with the use of insulin pumps and insulin analogues extensively in the clinics. The success of this method that provides eating flexibility and increases quality of life in diabetics is dependent on how much the diabetic individual perceives the method. The first of the two major factors that affect this perception is the level of information of the health staff to teach the method and the time spared for the patient. The other factor is patient motivation. The carbohydrate counting method that offers a flexible eating regimen to diabetic individuals is a functional method to render the patient more conscious and active about his/her disease and treatment.

## Figures and Tables

**Table 1:** Patients characteristics.

	Kh counting group	Control group
Number of patients (*n*)	22	15
Gender (male/female)	(8/14)	(7/8)
Age (year)	29.18 ± 2.06	29.67 ± 2.32
DM duration (years)	11.00 ± 1.34	6.80 ± 1.74

**Table 2:** The progress of the metabolic markers of the patients in the carbohydrate counting group.

	Carbohydrate counting group (*n* = 22)		*P* values
	Pre-treatment baseline values	Values at 6 months	
TA (mmHg)			*P* = 0.065
Systolic	109.77 ± 5.24	122.04 ± 2.60	(*P* > 0.05)
Diastolic	74.09 ± 1.63	79.54 ± 0.72	*P* = 0.005
			(**P** < 0.01**)
Fasting blood glucose (mg/dL)	149.63 ± 17.65	160.40 ± 19.00	*P* = 0.630 (*P* > 0.05)
Post-prandial blood glucose (mg/dL)	174.59 ± 15.46	169.27 ± 8.05	*P* = 0.710 (*P* > 0.05)
HbA1c (%)	8.14 ± 0.48	8.00 ± 0.38	*P* = 0.699 (*P* > 0.05)
Fructosamine (mg/dL)	417.77 ± 22.56	404.90 ± 23.59	*P* = 0.525 (*P* > 0.05)
Uri acid (mg/dL)	3.43 ± 0.26	3.94 ± 0.30	*P* = 0.003 (**P** < 0.01**)
Total cholesterol (mg/dL)	174.50 ± 8.24	170.77 ± 6.41	*P* = 0.534 (*P* > 0.05)
Triglyceride (mg/dL)	79.04 ± 6.83	75.90 ± 5.87	*P* = 0.587 (*P* > 0.05)
HDL cholesterol (mg/dL)	71.18 ± 4.19	67.40 ± 3.44	*P* = 0.112 (*P* > 0.05)
LDL cholesterol (mg/dL)	84.49 ± 7.01	92.86 ± 6.01	*P* = 0.120 (*P* > 0.05)
Total cholesterol/HDL cholesterol ratio	2.52 ± 0.18	2.71 ± 0.18	*P* = 0.174 (*P* > 0.05)

**Table 3:** Values for total insulin dose in the carbohydrate counting group and the control group.

	Carbohydrate counting group (*n* = 22)	Control group (*n* = 15)	*P* values
Total insulin doses Pre-treatment baseline	55.22 ± 4.70	53.53 ± 5.73	*P* = 0.820 (*P* > 0.05)
Total insulin doses at 6 months of the trial	43.77 ± 3.05	46.20 ± 5.06	*P* = 0.666 (*P* > 0.05)

*P* value	*P* = 0.004 (*P* < 0.01**)	*P* = 0.326 (*P* > 0.05)	

**Table 4:** Quality of life results in the carbohydrate counting group.

	Carbohydrate counting group (*n* = 22)		*P* values
	Pre-treatment baseline values	Values at 6 months	
Overall health (general point of view)	55.13 ± 3.90	68.31 ± 2.42	*P* = 0.002 (**P** < 0.01**)
Change in health status within the last one year	63.63 ± 5.13	89.77 ± 2.68	*P* = 0.000 (**P** < 0.001***)
Physical function	89.77 ± 2.64	92.72 ± 1.96	*P* = 0.238 (*P* > 0.05)
Physical functionality	82.95 ± 5.03	94.31 ± 2.28	*P* = 0.047 (**P** < 0.05*)
Mental function	77.00 ± 5.58	92.77 ± 3.10	*P* = 0.022 (**P** < 0.05*)
Social function	84.40 ± 3.37	93.18 ± 2.55	*P* = 0.018 (**P** < 0.05*)
Pain	82.31 ± 3.45	80.18 ± 3.22	*P* = 0.629 (*P* > 0.05)
Mental health	59.09 ± 3.82	65.09 ± 2.59	*P* = 0.169 (*P* > 0.05)
Energy	53.63 ± 3.08	63.40 ± 2.72	*P* = 0.003 **(** **P** < 0.01**)

**Table 5:** Quality of life findings in the control group.

	Control group (*n* = 15)		*P* values
	Pre-treatment baseline values	Values at 6 months	
Overall health (general point of view)	47.66 ± 5.27	56.06 ± 3.97	*P* = 0.015 (**P** < 0.05*)
Change in health status within the last one year	53.33 ± 6.39	61.66 ±4.13	*P* = 0.238 (*P* > 0.05)
Physical function	85.33 ± 4.09	79.33 ± 6.01	*P* = 0.098 (*P* > 0.05)
Physical functionality	75.00 ± 7.71	73.33 ± 7.89	*P* = 0.865 (*P* > 0.05)
Mental function	70.80 ± 7.21	73.00 ± 6.71	*P* = 0.809 (*P* > 0.05)
Social function	67.70 ± 6.98	79.53 ± 5.78	*P* = 0.083 (*P* > 0.05)
Pain	71.33 ± 5.53	66.20 ± 6.51	*P* = 0.470 (*P* > 0.05)
Mental health	57.60 ± 4.37	57.33 ± 4.39	*P* = 0.953 (*P* > 0.05)
Energy	49.66 ± 4.81	54.33 ± 5.13	*P* = 0.416 (*P* > 0.05)
